# Severe forms of partial androgen insensitivity syndrome due to p.L830F novel mutation in androgen receptor gene in a Brazilian family

**DOI:** 10.1186/1756-0500-4-173

**Published:** 2011-06-06

**Authors:** Reginaldo J Petroli, Andréa T Maciel-Guerra, Fernanda C Soardi, Flávia L de Calais, Gil Guerra-Junior, Maricilda Palandi de Mello

**Affiliations:** 1 Centro de Biologia Molecular e Engenharia Genética (CBMEG), Universidade de Campinas (UNICAMP), Avenida Cândido Rondon 400, Campinas, 13083-875, SP, Brasil; 2 Departamento de Genética Médica, Faculdade de Ciências Médicas, Universidade de Campinas (UNICAMP), Rua Tessália Vieira de Camargo 126, Campinas, 13081-970, SP, Brasil; 3 Grupo Interdisciplinar de Estudos da Determinação e Diferenciação do Sexo (GIEDDS); Faculdade de Ciências Médicas, Universidade de Campinas (UNICAMP), Rua Tessália Vieira de Camargo 126, Campinas, 13081-970, SP, Brasil; 4 Departamento de Pediatria, Faculdade de Ciências Médicas, Universidade de Campinas (UNICAMP), Rua Tessália Vieira de Camargo 126, Campinas, 13081-970, SP, Brasil

## Abstract

**Background:**

The androgen insensitivity syndrome may cause developmental failure of normal male external genitalia in individuals with 46,XY karyotype. It results from the diminished or absent biological action of androgens, which is mediated by the androgen receptor in both embryo and secondary sex development. Mutations in the androgen receptor gene, located on the X chromosome, are responsible for the disease. Almost 70% of 46,XY affected individuals inherited mutations from their carrier mothers.

**Findings:**

Molecular abnormalities in the androgen receptor gene in individuals of a Brazilian family with clinical features of severe forms of partial androgen insensitivity syndrome were evaluated. Seven members (five 46,XY females and two healthy mothers) of the family were included in the investigation. The coding exons and exon-intron junctions of androgen receptor gene were sequenced. Five 46,XY members of the family have been found to be hemizygous for the c.3015C>T nucleotide change in exon 7 of the androgen receptor gene, whereas the two 46,XX mothers were heterozygote carriers. This nucleotide substitution leads to the p.L830F mutation in the androgen receptor.

**Conclusions:**

The novel p.L830F mutation is responsible for grades 5 and 6 of partial androgen insensitivity syndrome in two generations of a Brazilian family.

## Findings

The androgen insensitivity syndrome (AIS, OMIN #300068) is a recessive disorder linked to the X chromosome. It may result in the failure of external genitalia masculinization in individuals with 46,XY karyotype and normal androgen production and metabolism [1,2]. There is a wide range of clinical manifestation, therefore the syndrome can be divided in three subgroups according the degree of undermasculinization: 1) mild AIS (MAIS) that is characterized by gynecomastia and infertility in phenotypically male individuals; 2) partial AIS (PAIS) that may present with predominantly male development or ambiguous genitalia (AG) or even with predominantly female external genitalia with clitoromegaly and/or posterior labial fusion and breast and pubic hair development; 3) complete AIS (CAIS) resulting in female external genitalia, sparse to absent pubic and axilary hair and normal breast development [2-6]. Due to significant differences generally found among clinical manifestation features in PAIS, some authors assigned grades ranging from 1 to 6 to describe patients that presented with different phenotypes varying from male genitalia and infertility to female genitalia with pubic and underarm hair [2,5,6].

The androgen activity is mediated by the androgen receptor (AR), a member of nuclear receptor family, which is encoded by the androgen receptor gene (*AR*). The gene is located on the X-chromosome at Xq11 - 12 and is formed by eight exons and seven introns that spans ~ 90 kb of DNA [7,8]. The AR protein contains approximately 919 amino acid residues, but this number is variable due to the existence of both polyglutamine and polyglycine stretches in the amino terminal region that may vary in length conferring normal variability among individuals [9,10]. Like other members of the nuclear receptor superfamily, the AR contains four different functional domains: an amino-terminal domain encoded by exon 1, which is a non-conserved region involved in transcriptional activation of target genes [11]; a central DNA-binding domain (DBD) encoded by exons 2 and 3, which contains two zinc finger motifs [12,13]; a hinge region containing the nuclear targeting signal [14] and a C-terminal ligand binding domain (LBD) encoded by exons 4-8 that also encompasses sub-domains involved in dimerization and transcriptional activation processes [15,16].

Mutations in the *AR *gene lead to AIS [5,17,18]. Such mutations are found differently distributed throughout the gene sequence [[19], website: http://www.mcgill.ca/androgendb]. Almost 70% of 46,XY affected individuals inherited mutations from their carrier mothers [20]. As discussed by Boehmer *et al. *[21] the identification of a specific AR mutation and its residual androgen action always provide more precise diagnosis and/or prognosis, which might contribute to the decision for sex assignment of 46,XY individuals with AIS and facilitate genetic counseling of carrier females. In addition, due to clinical and genetic heterogeneity of the condition, studies describing novel mutations in AIS provide important information for the function of a specific amino acid residue.

Therefore, the purpose of this study was to identify the *AR *gene mutation in a Brazilian family with five patients presenting PAIS corresponding to grades 5 and 6. The role of the novel p.L830F missense mutation in the AR within LBD is discussed by comparing structural characteristics of both normal and mutant proteins.

## Ethics and Consents

**Figure 1:**
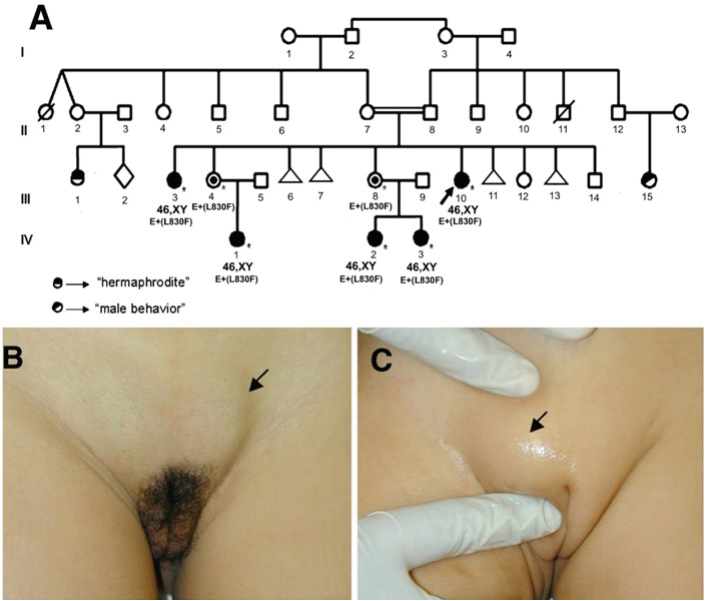
**Inheritance of androgen insensitivity syndrome in the family**. Family pedigree (A). Female genitalia with palpable gonads in the index case III-10 (B) and her niece IV-1 (C).

This study was approved by the Ethics Committee from Universidade Estadual de Campinas (São Paulo, Brasil) and informed consents were obtained from patients and relatives; informed consents from individuals III-10 and IV-1 were obtained separately for the publication of Figures 1B and 1C.

## Methods

Seven individuals from two generations of a family (Figure 1A) were included in this study. The index case (Figure 1A, III-10), an 18-year-old girl, was referred to us due to primary amenorrhea with spontaneous telarche and pubarche and palpable gonads in the inguinal region. She was born at term after an uneventful pregnancy by cesarian section with a birth weight of 3,330 g and height of 48 cm. Bilateral inguinal gonads were detected at birth. She was followed up over a period of eight months when assignment of female gender was decided; corrective procedure for inguinal gonads was planned but did not occur. On physical examination, she had typical female external genitalia, with only a slight posterior fusion of labioscrotal folds. Palpable gonads were found bilaterally in the inguinal region with volumes of 15 cm^3^, whereas pubertal development had reached Tanner stage B4P5 (Figure 1B). The uterus was absent under pelvic sonograms. Hormonal evaluation revealed normal FSH (5.4 mIU/mL; normal range (NR): 1.5 - 12.4 mIU/mL) and elevated levels of both LH (21.2 mIU/mL; NR: 1.7 - 8.6 mIU/mL) and total testosterone (>15 ng/mL; NR: 2.86 - 8.1 ng/mL); her karyotype was 46,XY. The bone mineral density test revealed femoral osteopenia and lumbar osteoporosis. Gonadectomy was performed a few months later and histological analysis revealed bilateral testes with no evidence of malignancy. She was subsequently referred to other services to perform vaginoplasty and to start hormone replacement therapy with estrogens and treatment of osteopenia/osteoporosis. Her parents were first cousins, and there was a positive family history with individuals presenting similar features: three nieces with palpable gonads and an older sister.

A 24-year-old sister (Figure 1A, III-3) that had inguinal gonads corrected in the first year of life, referred with primary amenorrhea, spontaneous breast development. On physical examination, pubertal development had reached Tanner stage B5P4. She was oriented to perform bilateral gonadectomy, vaginoplasty and hormone replacement.

A 3-year old niece (Figure 1, IV-1) was the only child of unrelated parents. She was born at term by normal delivery after an uneventful pregnancy with birth weight of 2,800 g, and bilateral inguinal gonads were detected at birth. On physical examination, she had typical pre-pubertal female genitalia and bilaterally palpable gonads were found in the inguinal region (Figure 1C). Her karyotype was 46,XY. After receiving all relevant information about risks and benefits of early versus late gonadectomy, her parents decided to delay surgery in order to allow spontaneous puberty. The girl was then referred to the pediatric endocrinology service for follow up.

A 1.5-year-old niece (Figure 1A, IV-2) was also the first child of unrelated parents. She was born at term by normal delivery after an uneventful pregnancy with birth weight of 2,650 g, and palpable gonads in the inguinal region were detected at birth. On physical examination, she had typical female external genitalia, with only a slight posterior fusion of labioscrotal folds. Her karyotype was 46,XY. Her parents decided to delay surgery until after puberty and the girl was referred to the pediatric endocrinology service for follow up. Her sister (Figure 1A, IV-3) was brought to us when she was 3 month old due to palpable gonads in the inguinal region, which had been detected at birth. She was born at term after an uneventful pregnancy by normal delivery with a birth weight of 3,180 g and length 49.5 cm. On physical examination, she had typical female external genitalia and both gonads were palpable in the inguinal regions. Her karyotype was 46,XY.

Samples of genomic DNA were obtained from peripheral blood by proteinase K/phenol extraction method [22]. Molecular analysis was performed by amplifying the eight exons of *AR *gene using the polymerase chain reaction (PCR) followed by sequencing the fragments using Big Dye^® ^Terminator Cycle Sequencing Kit V3.1 Ready Reaction (ABI PRISM/PE Biosystems). The sequences obtained in an ABI 3700 Sequencer (ABI PRISM/PE Biosystems) were compared with the normal sequence of the gene (ENSEMBL-ENSG00000169083) using Chromas (reduced version - free software) and GeneRunner v.3.05 (free software) or CLC Sequence Viewer v.6.2 (free software).

The model of human AR mutant protein was built using the resolved 3-D structure of human AR (PDB accession # 2AM9) as template. Molecular modeling was performed using MODELLER web-server program. The model images were examined and edited using PyMOL^® ^program and Millennium STING (CNPTIA-Embrapa, Brasil). The human AR sequence was compared with the corresponding mammalian proteins sequences in the ClustalW http://www.genome.jp/tools/clustalw/.

## Results

**Figure 2:**
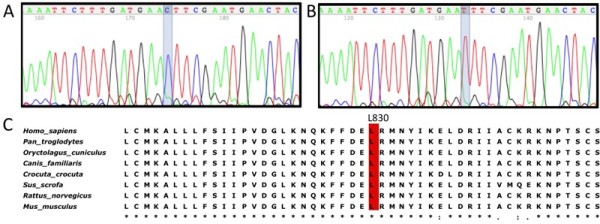
**Nucleotide change leading to p.L830F mutation and protein residue conservation**. The c.3015C>T nucleotide change in exon 7 is illustrated in the AR gene partial electropherogram: (A) normal sequence; (B) hemizygous mutant sequence from the index case III-10. (C) Part of multiple sequence alignment for the human AR protein and other mammalian AR proteins; the residue 830 is depicted in red for all species.

Upon sequencing exons 2 to 8 of the *AR *gene, a novel c.3015C>T nucleotide change in exon 7 was identified in five 46,XY female hemizygote individuals and also in two heterozygote carrier mothers (Figure 2A). This nucleotide change cause the putative replacement of a leucine by a phenylalanine residue at codon 830 (p.L830F).

Exon 1 sequencing showed 21 and 20 repeats of each CAG (SNP # rs5902610) and GGC, respectively, for the affected individuals. In addition, three GGT codons preceded the GGC stretch instead the two normally found in the *AR *gene. Besides the mutation, the heterozygote carrier mothers (Figure 1A: III-4, III-8) were also heterozygous for the c.639G>A (SNP # rs6152) and c.2319-78T>G (SNP # rs1337076) polymorphisms in exon 1 and intron 5, respectively.

**Figure 3:**
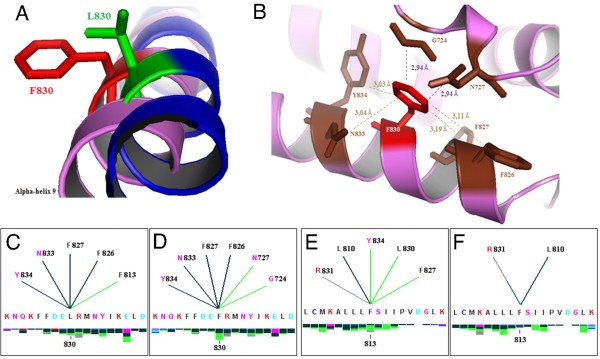
**Modeling for normal and mutant AR proteins**. (A) Comparison of normal and mutant human AR protein models at residue 830; normal leucine is denoted in green and phenylalanine in red. (B) Distances in Ångström (Å) for contacts for the F830 mutant residue estimated using PyMOL software: hydrogen bonds are shown in brown and hydrophobic interactions in purple. (C - F) Internal contacts provided by the analysis with BlueStar STING software. The native residue L830 (C) forms energetic hydrogen bonds with F826, F827, N833 and Y834 and a hydrophobic interaction with F813, whereas the mutant residue F830 (D) suppresses the interaction with F813 and introduces two additional hydrophobic interactions with G724 and N727. Effects upon internal contacts for F813 residue: in the normal protein, the F813 residue presents hydrophobic interaction with F827 and Y834 (E), whereas in the mutant protein those interactions are lost (F).

Multiple alignments comparing the human AR protein sequence to other mammalian AR proteins indicated the L830 as a highly conserved residue (Figure 2B). The structural analysis by modeling normal and mutant proteins (Figure 3A) revealed that the discrepancy of the mutant F830 compared to the normal L830 resides mainly in the abolishment of a hydrophobic interaction with F813 residue and in the creation of two different internal hydrophobic interactions with both G724 and N727 residues (Figure 3B and 3C). Since the interaction between L830 and F813 residues was suppressed by the mutation, several interactions involving F813 and other amino acids have also been disrupted (Figure 3D). It was also observed that the distance between F830 and either G724 or N727 residues has shortened to 2.94 Å (Figure 3B).

## Discussion

We report here the novel p.L830F mutation in the hormone binding region of the androgen receptor that is responsible for partial androgen insensitivity syndrome in a Brazilian family. Five patients in two generations carry the mutation. The index case (Figure 1A, III-10) and her niece (Figure 1A, IV-2) showed clinical and laboratorial data compatible with PAIS grade 5, since they had female external genitalia with a slight posterior fusion of labioscrotal folds and palpable gonads [2,6]. Whereas, her older sister (Figure 1, III-3) and two nieces (Figure 1, IV-1 and IV-3) presented with typical female external genitalia and palpable gonads [2,6], which classify them as PAIS grade 6. The presence of pubic hair was observed in the two sisters; however, it could not be verified in the nieces because they have not reached puberty yet.

In addition to the mutation, all patients presented 21 and 20 repeats for polyglutamine and polyglycine stretches, respectively, both within the range described as normal [23]. The 46,XX heterozygote mothers (Figure 1: III-4, III-8) were also heterozygous for both c.639G>A (SNP # rs6152) and c.2319-78G>T (SNP # rs1337076) polymorphisms. Since A and T nucleotides are, respectively, rare and very rare alleles (NCBI SNP database) in Caucasian populations it can be conclude that the paternal inherited allele also corresponds to a rare *AR *allele.

Missense mutations in AR protein may cause a spectrum of phenotypes that include complete androgen insensitivity in 46,XY individuals with female genitalia and partial androgen insensitivity in 46,XY individuals with male phenotype, except for perineoscrotal hypospadias, gynecomastia and/or infertility [19]. The phenotype variability appears to reflect the degree to which ligand-binding and receptor functions are disrupted by different substitutions [24]. In addition, genetic background also influences the resulting phenotype since a same mutation may cause different forms of AIS within a family [3,25]. The most frequent are missense mutations that are found within two important areas of the receptor protein: DBD and LDB domains [8]. The importance of the L830 residue for the AR activity may be inferred by p.L830V described before in a patient with CAIS [26]. In addition, the neighboring codon 831 was target for several missense mutations such as p.R831L and p.R831Q also causing CAIS [27,28]. Therefore, mutations in codon 830 may severely disturb the functional activity of LBD in which two hot spots for mutations have been identified [24].

X-ray crystallographic studies showed that the three-dimensional structure of the AR-LBD encompasses 12 α-helices [29]. In the normal molecule these α-helices undergo conformational changes in response to ligand-binding resulting in the assembly of the AF-2 domain. According to AR structure, the amino acid L830 is located within the α-helix 9 that comprises residues 825-847. This region has been proposed as part of an allosteric regulatory site termed binding function 3 (BF-3) where ligand interactions exert indirect effects on AF-2 to modulate co-regulator binding [30]. Mutations in BF-3 have demonstrated to diminish AR activity and they can be related with different phenotypes [30].

When the leucine is replaced by the phenylalanine in codon 830 the hydrophobic character of the residue is maintained, but the change of a high hydrophobic leucine to a less hydrophobic phenylalanine might affect the transcriptional activity as described for p.F826L mutation [31]. Considering the structural analysis for p.L830F, it can be proposed that the suppression of a hydrophobic interaction with residue F813 might destabilize the interaction between alpha-helices 8 and 9 which forms an important hydrophobic core to keep the AR binding capacity [32]. Conversely, the mutant residue established novel contacts with amino acids G724 and N727 that are located between alpha-helices 3 and 4, both residues are highly conserved and critical for ligand binding [17,31,33]. The creation of such hydrophobic contacts suggests that the p.L830F substitution would reduce the mobility of the region involving alpha-helix 9 and the region between alpha-helices 3 and 4. Also, those new interactions brought F830 and each G724 and N727 amino acids more close such as surface contacts of F830-G724 and F830-N727 enhanced leading to stronger interactions between the loop region located in the middle of alpha-helices 3 and 4 and alpha-helix 9. Those interactions give the region less flexibility than that necessary to allow properly co-factor assembly of AF-2 domain [34]. In conclusion, the decrease on the hydrophobicity of residue 830 and changes in the internal contacts caused by p.L830F mutation are probably responsible for a very low androgen receptor activity which might correlate with the severe PAIS phenotype observed for the patients. Similarly to other mutations described in this domain as causing different phenotypes within a family [3,25], p.L830F produced different PAIS grades in the family described here indicating an influence of genetic background on its effect.

## Competing interests

The authors declare that they have no competing interests.

## Authors' contributions

RJP and FLC carried out sequencing experiments and sequence alignment analysis; FCS contributed with the protein structural analysis; ATMG and GGJ were responsible for diagnosis and management of patients and participated in the design of the study; MPM conceived the study, and participated in its design and coordination and also drafted the manuscript. All authors read and approved the final manuscript.
